# Extra-cardiac findings in cardiovascular magnetic resonance: what the imaging cardiologist needs to know

**DOI:** 10.1186/s12968-016-0246-1

**Published:** 2016-05-09

**Authors:** Jonathan C. L. Rodrigues, Stephen M. Lyen, William Loughborough, Antonio Matteo Amadu, Anna Baritussio, Amardeep Ghosh Dastidar, Nathan E. Manghat, Chiara Bucciarelli-Ducci

**Affiliations:** Cardiovascular Magnetic Resonance Unit, NIHR Bristol Cardiovascular Biomedical Research Unit, Bristol Heart Institute, University of Bristol, Bristol, UK; Department of Clinical Radiology, Bristol Royal Infirmary, University Hospitals Bristol NHS Foundation Trust, Upper Maudlin Street, Bristol, BS2 8HW UK; School of Physiology, Pharmacology and Neurosciences, Faculty of Biomedical Sciences, University of Bristol, Medical Sciences Building, University Walk, Bristol, BS8 1TD UK; Department of Surgical, Microsurgical and Medical Sciences, Institute of Radiological Sciences, University of Sassari, Sassari, Italy; Department of Cardiology, Bristol Royal Infirmary, University Hospitals Bristol NHS Foundation Trust, Upper Maudlin Street, Bristol, BS2 8HW UK

**Keywords:** Extra-cardiac findings, Extra-cardiac pathology, Incidental findings, Cardiovascular Magnetic Resonance, CMR

## Abstract

Cardiovascular magnetic resonance (CMR) is an established non-invasive technique to comprehensively assess cardiovascular structure and function in a variety of acquired and inherited cardiac conditions. A significant amount of the neck, thorax and upper abdomen are imaged at the time of routine clinical CMR, particularly in the initial multi-slice axial and coronal images. The discovery of unsuspected disease at the time of imaging has ethical, financial and medico-legal implications. Extra-cardiac findings at the time of CMR are common, can be important and can change clinical management. Certain patient groups undergoing CMR are at particular risk of important extra-cardiac findings as several of the cardiovascular risk factors for atherosclerosis are also risk factors for malignancy. Furthermore, the presence of certain extra-cardiac findings may contribute to the interpretation of the primary cardiac pathology as some cardiac conditions have multi-systemic extra-cardiac involvement. The aim of this review is to give an overview of the type of extra-cardiac findings that may become apparent on CMR, subdivided by anatomical location. We focus on normal variant anatomy that may mimic disease, common incidental extra-cardiac findings and important imaging signs that help distinguish sinister pathology from benign disease. We also aim to provide a framework to the approach and potential further diagnostic work-up of incidental extra-cardiac findings discovered at the time of CMR. However, it is beyond the scope of this review to discuss and determine the clinical significance of extracardiac findings at CMR.

## Background

**Fig. 1:**
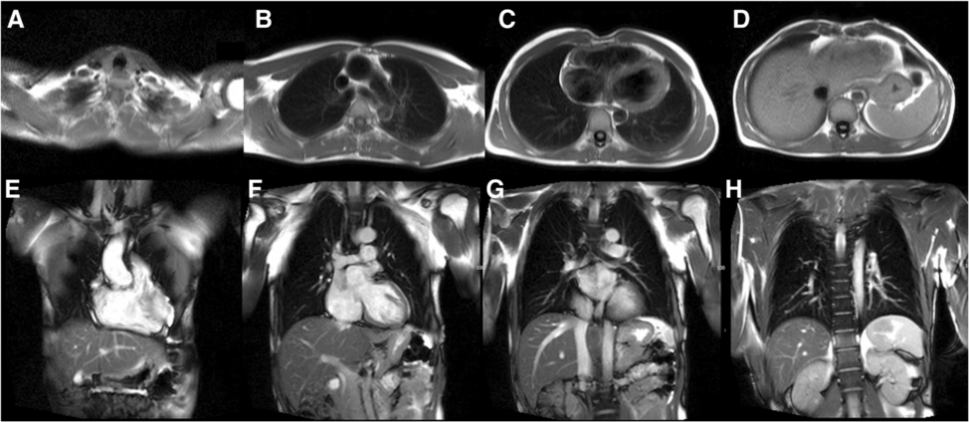
**a** to **d** Examples of the routine axial black blood images of the neck, thorax and upper abdomen acquired at the time of CMR (Cranial to Caudal). **e** to **h** Examples of the routine coronal bright blood images of the neck, thorax and upper abdomen acquired at the time of CMR (Anterior to Posterior)

**Fig. 2:**
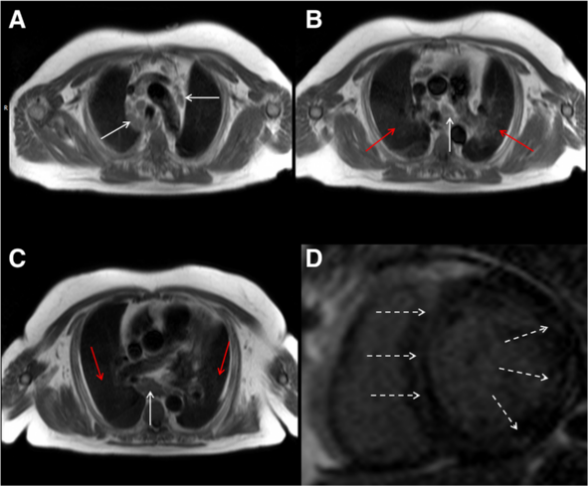
CMR performed in a patient with bifasicular block and transient 2:1 second degree atrio-ventricular block. Short-axis LV cine images revealed global LV systolic impairment with an ejection fraction of 31 %. There is evidence of aorto-pulmonary (**a**), para-tracheal (**a** and **b**) and subcarinal (**c**) lymphadenopathy (solid white arrows) on axial black blood imaging. There is also subtle parenchymal lung infiltrate (solid red arrows) in the midzones (**b** and **c**) on axial black blood images. The short axis magnitude late gadolinium enhancement images (**d**) reveal septal mid-wall and patchy lateral wall replacement fibrosis (dashed white arrows). Together, the cardiac and extra-cardiac findings, in the clinical context are consistent with cardiac and pulmonary sarcoidosis

Cardiovascular magnetic resonance (CMR) is an established non-invasive technique to comprehensively assess cardiovascular structure and function in a variety of acquired and inherited cardiac conditions [1–4]. Although the key components of a CMR study are acquired in specific imaging planes orientated along the axes of the heart, initial axial and occasionally coronal images of the thorax and upper abdomen are also acquired to help plan the study. Inevitably, this leads to imaging a significant volume of the patient beyond the target organ of interest, which may reveal extra-cardiac pathology (Fig. 1). A recent systematic review and meta-analysis of 12 studies including data from 7,062 patients demonstrated a pooled prevalence of incidental extra-cardiac findings of 35 %, with major incidental extra-cardiac findings in 12 % [5]. Extra-cardiac findings resulted in a change in management in 1 % of patients undergoing CMR [5], which is of particular importance to large volume CMR centres. The clinical use of CMR is growing year upon year, and the burden of extra-cardiac findings identified at the time of CMR is only likely to get larger as we image older individuals with multiple comorbidities. Furthermore, a large proportion of patients undergo clinical CMR for the assessment of ischaemic heart disease and some of the risk-factors for atherosclerosis (e.g. smoking, obesity, physical inactivity) are also recognized risk-factors for a variety of malignancies. The discovery of unsuspected disease at the time of imaging has ethical, financial and medico-legal implications [6–8]. However, given the incidental nature of the findings and the consequent non-dedicated imaging and tissue characterization, not infrequently these findings require additional confirmatory imaging. Furthermore, the presence of certain extra-cardiac findings can significantly contribute to the interpretation of the primary cardiac pathology as some cardiac conditions have multi-systemic involvement. For example, the discovery of mediastinal adenopathy and interstitial lung disease in an appropriate anatomical distribution would increase the likelihood of cardiac findings suggestive of cardiac sarcoidosis (Fig. 2). Depending on the institution, CMR examinations may be reported by Cardiologists, Radiologists or a combination thereof. Regardless of the specialty of the reporting doctor, it is clear that proper interpretation of clinical CMR studies requires a thorough understanding of cross-sectional anatomy and familiarity with normal anatomical variants, common and important pathology in the organs beyond the heart captured within the field of view of the CMR study. The importance of incidental non-cardiovascular findings has been recognized in the recent update to the European Association of Cardiovascular Imaging core syllabus for the European CMR certification exam [9, 10]. The key topics in the syllabus are detailed in Table 1.

**Table 1:** 2013 EACVI Core Syllabus for European CMR certification examination – adapted from references [9] and [10]

9. Incidental (non-cardiovascular) findings	
9.1. Neck including normal anatomy	
1/ Thyroid nodules	
2/ Adenopathy	
9.2. Thorax including normal anatomy	
1/ Lung (airspace disease, mass)	
2/ Pleura (effusion, neoplasm)	
3/ Mediastinum	
▪ Oesophagus (hernia, mass, dilatation/thickening)	
▪ Solid & cystic masses, adenopathy	
4/ Bone (fracture, neoplasm, infection)	
5/ Chest wall mass (breast, axilla)	
9.3. Abdomen including normal anatomy	
1/ Liver (cyst/haemangioma, mass, parenchymal disease)	
2/ Kidney (cyst, mass, hydronephrosis, parenchymal disease)	
3/ Adrenal mass	
4/ Spleen (size, lesion)	
5/ Other	
▪ Gallstones/cholecystitis	
▪ Ascites	

A variety of studies have previously explored the prevalence and clinical relevance of extra-cardiac findings. However, the aim of this review is to give an overview of the type of extra-cardiac findings that may become apparent at CMR, subdivided by anatomical location. We focus on normal variant anatomy that may mimic disease, common incidental extra-cardiac findings and important imaging signs that help distinguish sinister pathology from benign disease. We also aim to provide a framework to the approach and potential further diagnostic work-up of incidental extra-cardiac findings discovered at the time of CMR. This review cannot possibly equip Cardiologists who report CMR with specialist radiology training and is not a substitute to seeking expert Radiology input where necessary, but simply aims to provide an overview of some of the elements of radiological interpretation of extra-cardiac findings to try to avoid their misinterpretation, or missing them all together.

## Neck

Localiser images and cross sectional anatomical sequences, e.g. axial black blood imaging, will often demonstrate a portion of the neck and superior mediastinum. The main organ of interest imaged in this region in terms of extra-cardiac findings is the thyroid gland. It is an important organ because thyroid abnormalities are common. Supraclavicular and cervical adenopathy may also be demonstrated and the topic of pathological lymph nodal disease is discussed in the *Mediastinum* section of this review.

### Normal variant anatomy

The thyroid gland usually has two lobes joined in the midline by the isthmus. An additional pyramidal lobe can arise superiorly from the isthmus and, if present, is usually located deep to the medial border of the strap muscles of the neck, overlying laryngeal cartilage [11]. It should not be misinterpreted as cervical adenopathy.

### Common pathology

The most common thyroid pathology is the diffuse goitre. Its prevalence is greatest in pre-menopausal women and has a female preponderance (women: men = 4:1) [12] but prevalence decreases with age. On the contrary, the frequency of thyroid nodules increases with age, with the prevalence of clinically apparent thyroid nodules demonstrated in 6.4 % of women and 1.5 % of men aged >60 years old in a study of 5234 subjects [13]. Both these thyroid pathologies have been described in previous studies investigating the prevalence of extra-cardiac findings at the time of CMR [14–16].

### Important imaging findings

**Fig. 3:**
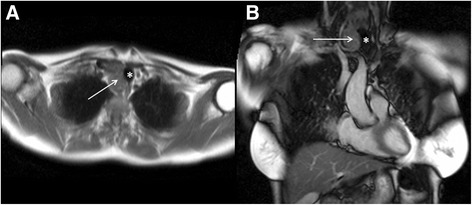
Axial black blood imaging of the neck (**a**) and coronal bright blood imaging (**b**) demonstrate homogeneous enlargement of the right lobe of thyroid gland (solid white arrows). There is local mass effect on the trachea with a degree of contralateral displacement and minor tracheal narrowing (*)

Whenever thyroid enlargement is detected, interrogating the available images for evidence of local mass effect is important. The position and calibre of the trachea should be assessed. Tracheal deviation (Fig. 3) and tracheal compression are important findings to mention in the clinical report. Indeed, they may even be implicated in the patient’s symptoms if they suffer from breathlessness. Defining the inferior extent of the enlarged thyroid gland is important. Significant retrosternal extension of the thyroid should be mentioned in a report as it hinders further evaluation of that portion of the thyroid with ultrasound, which is often the modality of choice for thyroid pathology.

The main concern regarding an incidental thyroid lesion at the time of CMR is whether it represents malignant disease. However, the images of the neck acquired at the time of CMR are usually very limited preliminary images and it is often not possible to answer this question. One of the reasons why magnetic resonance imaging (MRI) is unable to distinguish benign from malignant disease is because the spatial resolution of the images is insufficient to resolve some of the features associated with malignancy that may be demonstrated by ultrasound (e.g. micro-calcifications, intra-lesional vascularity and lobulated / irregular margins) [17]. Furthermore, both malignant and benign thyroid nodules have overlapping tissue characteristics and may both result in iso-intense T1 weighted and hyper-intense T2 weighted signal return [18]. Although nodule size only has a limited correlation with malignant potential, thyroid malignancies <2 cm have been reported to have a 99.9 % 10-year survival rate and failure to diagnose these lesions is unlikely to affect morbidity and mortality [19, 20]. MRI may underestimate the size of thyroid nodules relative to ultrasound, but it is reasonable to use MRI-derived measurements in risk stratification [21].

### Further investigations

The most recent American College of Radiology White Paper on the management of incidental thyroid nodules detected at MRI advises, in the absence of suspicious ancillary findings such as abnormal local lymph nodes and/or invasion of local tissues by the thyroid nodule, a cut-off of ≥1 cm in subjects <35 years old as a threshold to further investigation with ultrasound and a cut-off of ≥1.5 cm in subjects ≥35 years old [17]. The imaging modality of choice for further characterization is dedicated ultrasound, which affords the opportunity of simultaneous fine needle aspiration (FNA) as appropriate. Suggesting FNA correlation purely on the basis of the MRI images should be avoided because, although FNA is a minimally invasive procedure, the inability of cytology to definitively establish a benign diagnosis in a proportion of non-malignant nodules will expose a substantial number of patients to repeat biopsy or even lead to surgical excision of benign thyroid disease due to indeterminate cytological results [17].

Interpreting incidental thyroid lesions found at CMR may require liaising with the referring clinician to identify any specific thyroid disease risk factors (e.g. family history of thyroid or endocrine malignancy, previous neck irradiation etc.). Reviewing any previous ultrasound imaging and cross-sectional imaging is good practice and, if any diagnostic doubt, seeking the opinion of a Head and Neck Radiologist would be prudent.

## Mediastinum

Superior, anterior, middle and posterior components of the mediastinum are visualized on the axial cross-sectional sequences usually acquired to plan the subsequent specific cardiac cines and images.

### Normal variant anatomy

**Fig. 4:**
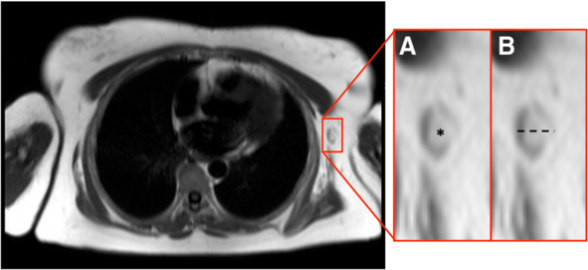
Axial black blood imaging of the thorax demonstrating a morphologically normal, ovoid left axillary lymph node (**a**) with fatty hilum (*). By convention, lymph node size (**b**) is measured in the short-axis (dashed black line)

Thoracic lymph nodes are a common extra-cardiac finding at CMR [5]. They are readily identified as discrete ovoid areas of soft tissue signal return within thoracic fat. Normal lymph nodes have characteristic morphology with a fatty hilum (Fig. 4a). When measuring lymph node dimensions, by convention, the short-axis diameter should be quoted and not the maximum dimension of the node (Fig. 4b). Generally, the upper limit of normality for mediastinal lymph nodes in terms of size is 10 mm in short-axis diameter [22–25]. However, there is variation in thoracic lymph node size depending on location, with a tendency for upper paratracheal nodes to be smaller at 7-8 mm [22, 23, 26]. Retrocrural and peridiaphragmatic nodes normally measure up to 6 mm and 5 mm respectively [27, 28]. However, it should be remembered that the axial black blood imaging often has a slice thickness of >5 mm, which may preclude accurate nodal size measurements.

**Fig. 5:**
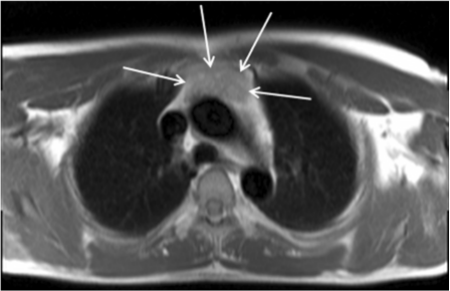
Axial black blood imaging of the superior mediastinum in a 20 year old male patient who had recently suffered from a severe pneumonia complicated with myocarditis. The anterior mediastinal soft tissue (solid white arrows) detected at the time of CMR was felt too prominent to be dismissed as normal residual thymus and given the clinical history the diagnosis of thymic reactivation was made

The thymus gland is a normal anterior mediastinal structure in children and young adults that should not be mistaken for an abnormal anterior mediastinal mass. It has a characteristic bi-lobed shape and convex margins which become concave with age [29]. Over time, the thymus gland undergoes fatty involution and is absent on imaging in 50 % of patients over 40 years old [30]. Thymic reactivation (Fig. 5) can occur following severe illness and may account for apparent normal thymic tissue in older individuals. However, in this scenario a thymic malignancy also needs to be considered.

The pericardial recesses are important mimics of lymphadenopathy and distinguishing characteristics include fluid signal, a beak-like shape, lack of mass effect and contiguity with the pericardium [31].

Comprehensive discussion of normal variant cardiovascular anatomy is beyond the scope of this review but individuals reporting CMR studies should be familiar with normal anatomical variations in cardiac position and situs, aortic arch anatomy, aberrant superior mediastinal arterial anatomy and anomalous systemic and pulmonary venous drainage.

### Common pathology

**Fig. 6:**
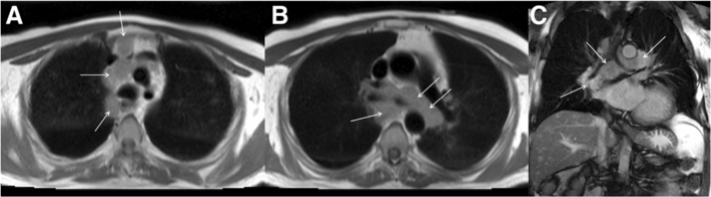
Axial black blood imaging (**a** and **b**) and coronal bright blood imaging (**c**) demonstrating florid mediastinal adenopathy engulfing the trachea and proximal main bronchi (solid white arrows) detected incidentally at the time of CMR

Thoracic lymphadenopathy is a common extra-cardiac finding at the time of CMR. Numerous pathologies can cause thoracic lymphadenopathy and if identified it should prompt a comprehensive review for the potential aetiology. Causes of benign lymph node enlargement include pulmonary infection, congestive heart failure [32], pulmonary fibrosis [33] and pneumoconioses [34]. Bilateral symmetrical hilar and mediastinal node enlargement in a young adult is most often due to sarcoidosis, although lymphoma is an important differential diagnosis. Lymphoma often involves the mediastinum and can coalesce to form large confluent mediastinal masses [35] (Fig. 6). Malignancies that commonly metastasize to the thoracic lymph nodes include lung, breast, head and neck, melanoma, gastrointestinal and genitourinary carcinomas [36] and evidence for these may be present on extracardiac images or preceding investigations.

**Fig. 7:**
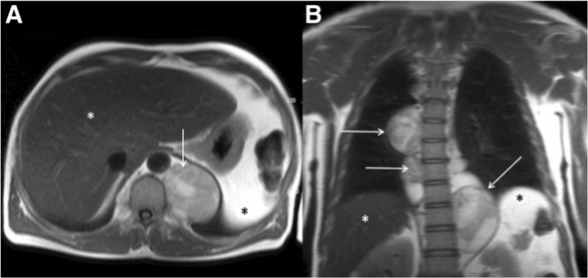
Axial black blood imaging (**a**) and coronal bright blood imaging (**b**) acquired during a myocardial and hepatic iron loading protocol CMR in an individual with thalassaemia major. There are several prominent heterogeneous posterior mediastinal masses (solid white arrows) consistent with extramedullary haematopoeisis. The liver is uniformly low signal (white *), which is visual evidence of hepatic iron loading in the clinical context and the spleen is absent (black *) which may be the result of an auto-splenectomy

Mediastinal masses are an uncommon but important consideration when reviewing the extra-cardiac structures, and if detected a Radiological opinion should be sought. Common anterior mediastinal neoplasms include thymoma, lymphoma or teratoma. The most common posterior mediastinal masses include lymphadenopathy and neurogenic tumours such as schwannomas and neurofibromas [37]. Extramedullary haematopoeisis is relatively uncommon but may be seen more frequently in centres where T2* MRI is used for assessment of myocardial and hepatic iron loading in, for example, thalassaemia patients (Fig. 7). A frequent incidental finding that may mimic an inferior posterior mediastinal mass is a hiatus hernia. This can be readily identified as a structure contiguous with the alimentary tract, on initial axial and/or coronal images, and may contain a gas/fluid level. Acid reflux from a hiatus hernia can sometimes be responsible for the symptoms in patients referred for investigation of atypical angina and, particularly in the case of a negative stress CMR test, it should be excluded.

### Important imaging findings

The most common dilemma when reviewing the mediastinum on any imaging modality is trying to determine whether the discernible lymph nodes are pathological. The loss of the normal ovoid shape, with a more rounded configuration, and obliteration of the normal fatty hilum are findings that suggest the lymph node is abnormal. The short-axis size cut-off values can be useful but it should be noted that there are limitations to applying size criteria alone to determine the presence of pathology, with studies showing relatively low sensitivity and specificity for detection of metastases [38, 39]. In addition, the lower spatial resolution in MRI may result in closely opposed lymph nodes being inseparable and appearing as a larger nodal mass [40].

### Further investigations

As with most imaging investigations, the next best investigation when faced with a mediastinal abnormality is to review the last investigation, when present. Increasing numbers of patients undergoing CMR have had previous cross-sectional imaging with computed tomography (CT) which can help determine the aetiology and significance of mediastinal abnormalities detected at the time of CMR. If in doubt, the expert opinion of a Radiologist should be sought.

## Lungs and pleura

The standard axial imaging performed at the time of CMR usually covers most of the thorax. MRI assessment of the lung parenchyma is often limited by a combination of respiratory motion artifact, low proton density of the aerated lung parenchyma and the presence of susceptibility artifact occurring at air-tissue interfaces [41]. Despite this, significant pulmonary abnormalities are found in up to 21.8 % of CMR examinations [5].

### Normal variant anatomy

**Fig. 8:**
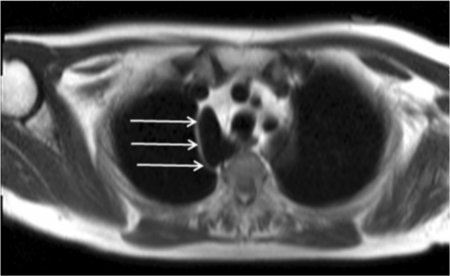
Axial black blood image of the superior mediastinum acquired at the time of CMR showing a linear high signal entity in the apical segment of the right upper lobe (solid white arrows) consistent with an incidental azygous lobe fissure

A common anatomical variant that should not be confused with pathology is the azygous lobe fissure (Fig. 8). It has been reported in 1 % of pathological specimens [42]. It occurs as the displaced azygous vein makes a deep invagination into the apical segment of the right upper lobe, carrying with it both pleural layers. As it lacks its own bronchus, it is not a true anatomical accessory lobe.

### Common pathology

Consolidation is not infrequently encountered. Technically, consolidation is a radiographic term referring to increase in lung parenchymal attenuation that obscures the vascular margins [43]. It is not specific to infection and can occur in pulmonary oedema, pulmonary haemorrhage and malignancy and they need to be differentiated on the basis of clinical findings.

**Fig. 9:**
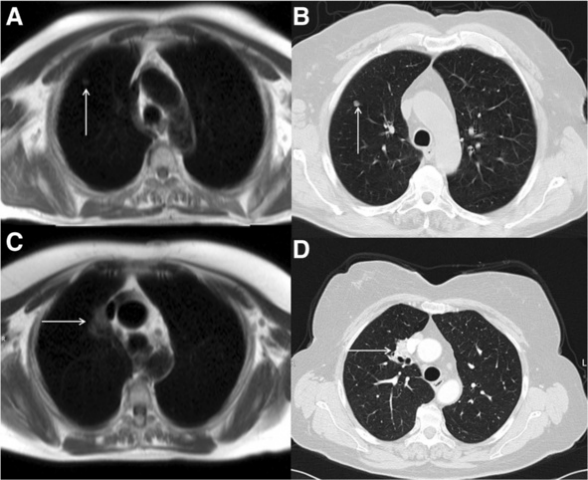
Axial black blood imaging (**a**) revealing an incidental 1.7 cm soft tissue density nodule in the right upper lobe (solid white arrows), which was subsequently confirmed with CT (**b**). Axial black blood imaging (**c**) demonstrating an incidental 3.1 cm soft tissue mass intimately related to the superior vena cava (solid white arrows), which was subsequently confirmed with CT (**d**)

Smoking is a risk factor for atherosclerosis and lung malignancy. Many subjects undergoing CMR are at risk of lung malignancy and the lungs should be reviewed carefully for lung nodules, defined as a rounded opacities well or poorly defined measuring ≤ 3 cm in diameter (Fig. 9a and b), and/or lung masses, defined as a opacity > 3 cm in diameter (Fig. 9c and d) [43]. It should be appreciated that, due to the slice thickness of the axial and coronal anatomical images, small nodules may not be discernible at the time of CMR.

**Fig. 10:**
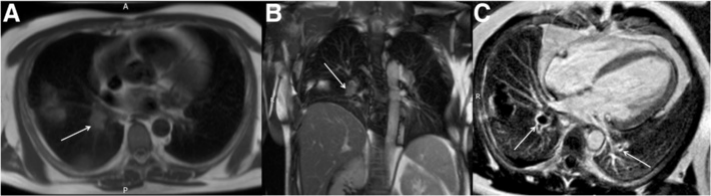
Axial black blood imaging (**a**) and coronal bright blood imaging (**b**) demonstrating abnormal signal in the right sided pulmonary arterial system (solid white arrows). Magnitude late gadolinium enhancement 4-chamber image (**c**) showing signal void in the pulmonary arteries (solid white arrows) consistent with in situ thrombus from pulmonary embolic disease

Pulmonary embolic disease is a common cause of chest pain but may also be detected incidentally. Dedicated MR pulmonary angiographic sequences can detect pulmonary emboli but are less specific than CT [44]. Nevertheless, pulmonary emboli may be detectable as abnormal signal return from the pulmonary arteries on standard axial black blood and coronal bright blood images (Fig. 10a and b). In addition, pulmonary emboli may be detectable on late gadolinium enhancement (LGE) images, with similar imaging appearances to ventricular thrombus but within the pulmonary arterial tree (Fig. 10c). Review for pulmonary emboli should be performed in patients undergoing CMR in the context of chest pain, elevated troponin but unobstructed coronary arteries where a pulmonary embolism may be the underlying aetiology but if there is clinical concern a CT pulmonary angiogram should be performed. However, if a pulmonary embolism is detected on CMR, the cine sequences should be reviewed for intracardiac thrombus and/or signs of right heart strain. Depending on the extent of the embolus, there may be associated pulmonary infarct or pleural effusion.

The most common pleural abnormality identified at the time of CMR is a pleural effusion. They are usually seen layered posteriorly with low T1 and high T2 signal, although this may be heterogeneous due to flow artefacts within.

### Important imaging findings

When faced with a lung parenchymal opacity, it is important to try to measure the maximal dimension of the entity. MRI has a sensitivity of up to 95 % for pulmonary nodules measuring 5-10 mm in size [45] and is as accurate as CT in detection of nodules over 1 cm in size [46]. Nodule size is the main predictor of malignancy. In the National Lung Screening Trial, the positive predictive value for malignancy of nodules 7-10 mm, 11-20 mm and 21-30 mm in size on CT were 1.7 %, 11.9 % and 29.7 % respectively [47]. The presence of spiculated margins is also suggestive of malignancy [48].

Although most pleural effusions encountered will be simple effusions, it is important to assess for features that would suggest a complex collection. The presence of loculation and pleural thickening are seen in exudative effusions. Pleural nodularity, circumferential or mediastinal pleural involvement are suggestive of malignancy [49]. Invasion into adjacent mediastinal or chest wall structures indicate aggressive infection or malignancy. Occasionally, pleural fluid may become encysted in the horizontal and/or oblique fissures and may mimic a mass but it should be readily differentiated on MRI to due fluid, rather than soft tissue, signal characteristics.

### Further investigations

When nodules are detected, a review of any previous imaging is essential to establish stability or if growth, over what time frame. Further evaluation with CT is often required and if appropriate the nodule can then be followed up as per the Fleischner Society guidelines [50] (Table 2) or the new 2015 British Thoracic Society guidelines for the investigation and management of pulmonary nodules [51]. If there is a suspicion of malignant nodule or mass, there should be a low threshold for seeking thoracic Radiology or Respiratory opinion. Urgent referral to the local lung cancer multidisciplinary meeting would be important in such cases.

**Table 2:** The Fleischner society recommendations for the follow-up and management of solid pulmonary nodules smaller than 8 mm detected incidentally in persons 35 years of age or older – adapted from reference [50]

Nodule size: ≤ 4 mm • Low risk^a^ : No follow-up • High risk^b^ : Follow-up CT at 12 months and if no change, no further follow-up required	
Nodule size: >4 – 6 mm • Low risk^a^ : Follow-up CT at 12 months and if no change, no further follow-up required • High risk^b^ : Initial follow-up CT at 6–12 months and then at 18–24 months if no change	
Nodule size: >6 – 8 mm • Low risk^a^ : Initial follow-up CT at 6–12 months and then at 18–24 months if no change • High risk^b^ : Initial follow-up CT at 3–6 months and then at 9–12 and 24 months if no change	
Nodule size: >8 mm • Follow-up CTs at around 3, 9 and 24 months or • Dynamic contrast enhanced CT, PET-CT, and/or biopsy	

^a^Low risk patients: minimal or absent history of smoking and / or other know risk factors

^b^High risk patients: history of smoking or other known risk factors

## Breast

The mammary tissue is visualized on axial cross sectional imaging at the time of CMR. Indeed, incidental breast lesions are identified in 0.1-2.5 % of CMR studies and over 50 % of these lesions are clinically significant [14–16, 52]. In addition, in women, breast cancer is the most frequently diagnosed cancer and the leading cause of cancer deaths on the worldwide scale [53]. However, there are increasing numbers of breast cancer survivors and there is a corresponding increase in cardiac imaging, sometimes with CMR, to assess for cardio-toxic side effects from some of the chemotherapeutic agents used to treat the disease. Particular care should be given to the breast tissue in CMR studies performed in such patients as they remain at risk of disease recurrence.

### Normal variant anatomy

Breast density varies with age with more fibro-fatty replacement of glandular tissue in the elderly. Occasionally, this process may be asymmetrical and a focus of persistent glandular tissue may mimic a breast lesion.

### Common pathology

**Fig. 11:**
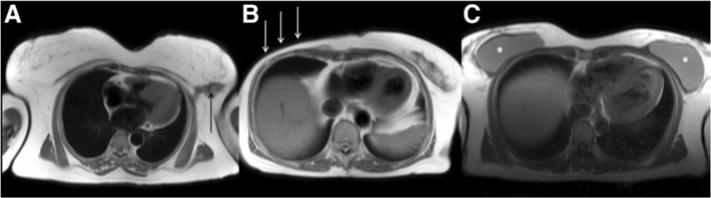
Axial black blood imaging (**a**) showing an abnormal, spiculate soft tissue signal mass in the fatty left breast (solid black lesion) detected incidentally at the time of CMR. Axial black blood imaging (**b**) showing previous right-sided mastectomy (solid white arrows). Axial black blood imaging (**c**) showing bilateral breast prostheses (*)

The differential diagnosis for a focal breast lesion (Fig. 11a) includes breast cyst, fibroadenoma, prominent area of fibrocystic change, fat necrosis, phyllodes tumour and, most concerning, malignancy. The age of the individual affects the likely aetiology. For example, breast cysts are a common cause in premenopausal women >40 years old but an infrequent cause of breast masses in younger women. Fibroadenomas are areas of overgrowth of normal breast tissue due to hormonal stimulation that are common in younger women.

Given the high prevalence of breast cancer, mastectomies are not infrequently encountered at CMR (Fig. 11b). Identifying such abnormality may have important implications as it implies a history of previous breast cancer, which may not always be stated in the clinical information accompanying the clinical CMR request. In such patients, the reporting doctor should have a heightened level of suspicion that lung and/or bony lesions could represent metastatic disease. Furthermore, lung parenchymal abnormality associated with volume loss in the ipsilateral upper zone may be a result of previous breast radiotherapy.

Breast implants, inserted for cosmetic or reconstructive purposes, are occasionally encountered (Fig. 11c). Implant rupture is a recognized complication and has received much media coverage in recent years. The high specificity of MRI at detecting features of rupture means those reporting MRI studies covering the breast need to be aware of the imaging features of rupture which can, albeit infrequently, be an incidental finding. The bright-blood, T2 weighted sequences are the most valuable in detecting potential rupture, which is classified as either intra- or extra-capsular. The most useful feature of an intra-capsular rupture is the ‘linguine sign’, which are curvilinear hypointense lines within the hyperintense silicon [54]. Focal invagination between the inner shell and fibrous capsule is termed the tear-drop sign [54]. Extra-capsular rupture is difficult to detect without breast dedicated MRI with silicon suppressed sequences.

### Important imaging findings

When an incidental breast lesion is discovered, the most important task is to try and positively identify malignant lesions. Certain loco-regional changes should be sought which increase the risk of malignancy, such as nipple inversion and cutaneous thickening. The lymphatic drainage of the breast is to the axillary, internal mammary and supraclavicular lymph node stations. Therefore, these nodal groups should be scrutinized in patients with known breast cancer and those where an incidental breast mass is identified. Nodal imaging features concerning for malignant infiltration include increased cortical thickness, soft tissue infiltration of the fatty hilum, lobulated shape and large size [55]. The size limits for abnormal lymph nodes depend on the sensitivity to specificity ratio desired. A short-axis diameter >5 mm has been demonstrated to be highly specific for malignant infiltration [56]. However, as previously discussed, determining pathological lymphadenopathy should not be made on size alone, but include morphological assessment too.

It should be noted that MRI is not a replacement for oncological staging of breast cancer and CT may still be required. Furthermore, normal breast appearance on MRI at the time of CMR does not exclude sinister breast disease and/or breast cancer recurrence.

### Further investigations

The information that can be gathered on the breast tissue from the routinely used CMR sequences is limited. The incidental breast mass cannot be fully characterized at the time of CMR and all lesions should be referred to a breast unit for further assessment, usually with dedicated clinical history, mammogram and/or ultrasound and tissue diagnosis as appropriate. Dedicated breast MRI is a specialized investigation involving the patient lying prone with dedicated breast coil and dynamic enhancement protocols and warrants specialized breast Radiologist interpretation.

## Liver, biliary tree and pancreas

Whilst the liver may only be partially imaged on the most inferior aspect of the field of view during dedicated cardiac MRI, it may be virtually completely imaged in whole aortic MRI sequences. Knowledge of normal anatomical variants and common hepato-biliary pathology is, therefore, important.

### Normal variant anatomy

**Fig. 12:**
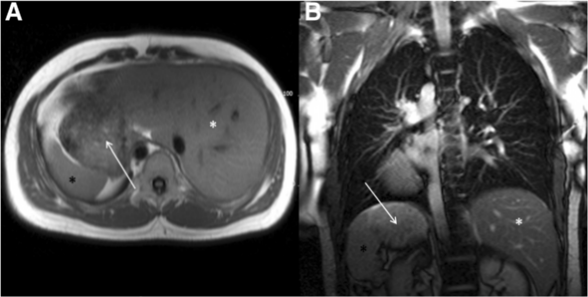
Axial black blood imaging (**a**) and coronal bright blood imaging (**b**) demonstrating a left-sided liver (white *), right-sided spleen (black *) and right-sided stomach (solid white arrow). There is also evidence of a right-sided descending thoracic aorta on the axial and coronal images

Individuals who regularly report congenital heart disease CMR will routinely assess the available images to determine abdominal situs, but all CMR imagers should be comfortable identifying such anatomical variations (Fig. 12).

Riedel’s lobe is an accessory right lobe of the liver. It is described as a tongue-like, inferior extension of the right lobe of liver into the right flank / right iliac fossa, beyond the right sided inferior costal cartilage [57]. It should not be confused with a hepatic mass or hepatomegaly.

### Common pathology

**Fig. 13:**
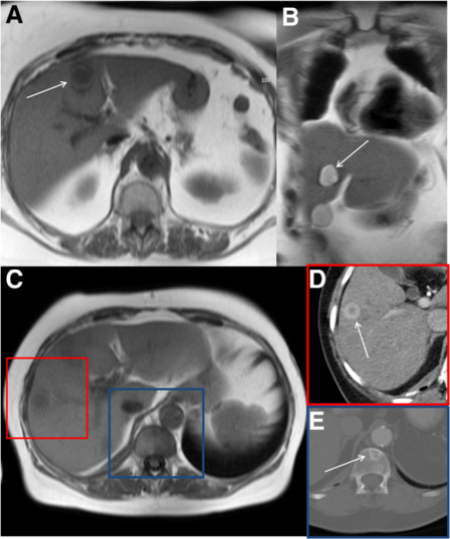
Axial black blood imaging (**a**) and coronal bright blood imaging (**b**) showing a well-defined low and high signal entity respectively consistent with a simple liver cyst. Axial black blood imaging (**c**) showing ill-defined low signal entity in the right lobe of liver (red box) which demonstrated features consistent with a metastasis on contrast enhanced CT (**d**). There is also a low signal entity in the vertebral body (blue box) which was confirmed to represent a sclerotic metastasis on CT (**e**)

Simple hepatic cysts are the most common hepatic parenchymal abnormality and are frequently encountered in the extra-cardiac field of view at the time of CMR. They will have high signal return on T2 weighted sequences (Fig. 13a and b). Not all cystic lesions in the liver are simple and the imaging differential diagnosis includes cystic tumours, parasitic or hydatid cystic disease, and hepatic abscesses. However, usually the ancillary clinical information enables the respective pathologies to be distinguished, with infective causes unlikely to be asymptomatic incidental findings.

Metastatic disease is the most common cause of malignant liver lesions (Fig. 13c and d). Colon, lung, breast and gastric malignancies have a predilection for metastasizing to the liver. The differential for focal liver parenchymal lesions includes hepatocellular carcinoma, adenomas, haemangiomas and focal nodular hyperplasia. Supportive clinical findings may be relevant in distinguishing the pathologies, for example hepatocellular carcinoma usually occurs in cirrhotic livers, whereas hepatic adenomas most frequently occur in young women on the oral contraceptive pill.

**Fig. 14:**
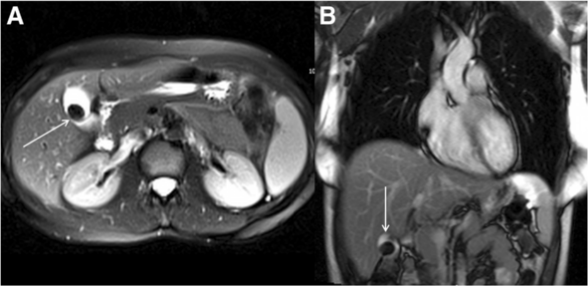
Axial (**a**) and coronal (**b**) bright blood imaging demonstrating a large low signal entity in a fluid-filled viscus in the right upper quadrant (solid white arrows), consistent with a large uncomplicated calcific gallstone

Gallstones occur in approximately 10-15 % of the adult population [58]. Calcified stones will appear as low signal entities on MRI (Fig. 14). Cholesterol stones may be high signal on T1 weighted images. However, not every individual with gallstone disease will be or will become symptomatic. Gallbladder wall thickening and pericholecystic fluid, suggesting cholecystitis, and biliary tree dilatation, raising the possibility of choledocholithiasis, are unlikely to be present when gallstone disease is detected incidentally at CMR, without right upper quadrant pain and liver function test derangement respectively. However, sometimes patients are referred for assessment of atypical chest pain, which has actually been caused by gallstone disease.

Images of the liver are routinely acquired when assessing for hepatic iron loading with MRI. Even when the dedicated gradient recall echo sequences required for assessment of liver T2* are not performed, visual assessment of the liver and spleen may provide useful insights into the aetiology of an individual with possible cardiomyopathy of indeterminate cause. The superparamagnetic effect of iron accumulation in the tissues results in local magnetic field distortion and shortening of T1 and T2 relaxation times. This in turn leads to signal loss from the affected organs. Diffuse low signal in both the liver and spleen raise the possibility of secondary cause of iron overloading, whereas low signal in the liver with preserved signal intensity in the spleen suggests primary hepatic iron deposition disease such as haemochromatosis.

The pancreas often undergoes fatty replacement with age and may appear atrophic. When a lesion is detected, the most concerning pathology to be excluded is pancreatic malignancy. When this occurs in the pancreatic head, there is often associated obstruction to the biliary tree and as a result it is unlikely to present incidentally. However, the differential diagnosis for focal pancreatic lesions is wide and if a lesion is identified then expert hepatobiliary radiology opinion is advised in the first instance to guide further investigation.

### Important imaging findings

In terms of extra-cardiac liver parenchymal lesions, the main concern is trying to distinguish liver metastases from benign liver lesions. Liver metastases are variable in their T1 and T2 signal intensities but usually result in hypo to iso-intense signal on T1 weighted images and iso to hyper-intense signal on T2 weighted images. Liver metastases tend to lose signal with increasing T2 weighting, whereas liver cysts and haemangiomas remain bright. However, metastases with necrotic cores can mimic cysts in terms of signal characteristics. Often it is not possible to definitively characterize liver lesions incidentally detected at the time of CMR.

### Further investigations

Reviewing any available previous liver imaging can be useful in determining the aetiology and necessity for further investigation of the lesion in question. Trans-abdominal ultrasound is a readily available, harmless investigation that is often recommended as the next investigation to try to characterize incidental liver lesions further. Entirely simple appearing liver cysts do not require further investigation. If there are any atypical features (e.g. ill-defined margins, complex structure, heterogeneous content) and there is no previous imaging for comparison then seeking the opinion of a Radiologist and referral for ultrasound should be considered. Comprehensive hepatic lesions characterization with MRI is a specialist investigation, which requires dedicated hepatic sequences and often necessitates specific contrast agents.

## Kidneys

Often only the upper poles of the kidneys are visualized due the constraints of the field of view at the time of CMR. This can make accurate localization and characterization of potential renal abnormalities challenging, which may lead to over-investigation, potential misinterpretation and incorrect diagnosis.

### Normal variant anatomy

The size of the kidneys in adult subjects is 10 – 12 cm. There is normally a slight discrepancy in the length of the kidneys, with the left kidney usually being slightly larger by approximately 1 centimetre. If the length of the patient’s kidneys is going to be measured, it is important that multiplanar reformatted images adjusted to give a true long-axis image of each individual kidney are used to avoid recording foreshortened measurements.

There are several anatomical variations in the kidneys that may mimic pathology. For example, it is not uncommon for the contour of the kidney to be slightly irregular. This may be simply due to persistent foetal lobulation or a dromedary hump, the latter is more common in the left kidney, rather than a parenchymal abnormality. In the case of anatomical variations, the cortico-medullary differentiation will be preserved and the cortical thickness uniform.

**Fig. 15:**
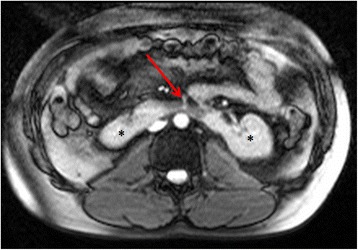
Axial bright blood imaging revealing a horseshoe kidney (*). The usual ascent of the kidneys out of the pelvis is impeded by the physical constraint of the inferior mesenteric artery (solid red arrow)

Occasionally, one or both kidneys are not demonstrated on the axial or coronal images at the time of CMR. Providing there is no preceding history of nephrectomy, this is often due to low-lying kidney(s) or pelvic kidney(s). Sometimes, the kidneys are fused in the midline and fail to ascend out of the lower abdomen due to the inferior mesenteric artery blocking the path. This is termed horseshoe kidney (Fig. 15) and is associated with certain congenital heart diseases.

Another renal anatomical variation that may cause confusion is the duplex kidney. This occurs when there is either complete or partial duplication of the renal collecting system within one kidney. In a complete duplex kidney, the 2 renal pelvicalyceal systems drain via separate ureters into the bladder. The upper moiety often inserts ectopically and may be associated with a distal ureterocoele resulting in urinary obstruction and hydronephrosis, whereas the insertion site of the lower moiety ureter is prone to urinary reflux, which may result in renal scarring.

### Common pathology

**Fig. 16:**
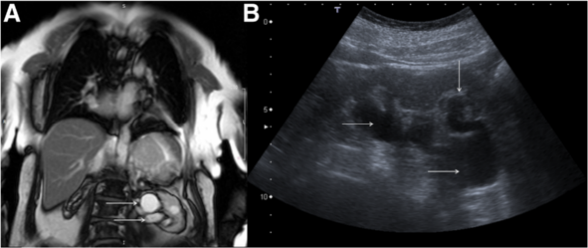
Coronal bright blood imaging (**a**) demonstrating a cystic entity at the left renal pelvis (solid white arrows). There is also a simple cortical renal cyst in the left lower pole. Correlative trans-abdominal ultrasound image (**b**) revealed the hypoechoic areas at the renal hilum are in continuity with a dilated proximal ureter and there is calyceal blunting (solid white arrows). These findings are consistent with hydronephrosis rather than para-pelvic cysts

The most common focal renal pathology identified at CMR is a renal cyst. Most of these lesions will be simple benign cysts of no clinical relevance. However, a proportion of cystic renal lesions have malignant potential. Sometimes the position of renal cysts can mimic other pathology, for example prominent para-pelvic renal cysts can sometimes be confused with hydronephrosis (Fig. 16).

It is not uncommon to detect global renal cortical atrophy, particularly in elderly subjects with cardiovascular risk factors. The patient’s renal function should be routinely checked before CMR and administration of intravenous gadolinium chelate contrast agent so the discovery of such renal appearances should not be unexpected to the referring clinician. Occasionally, unilateral renal atrophy will be demonstrated. In such cases, it is worth raising the question of ipsilateral renal artery stenosis as the cause, which may be amenable to intervention.

### Important imaging findings

**Fig. 17:**
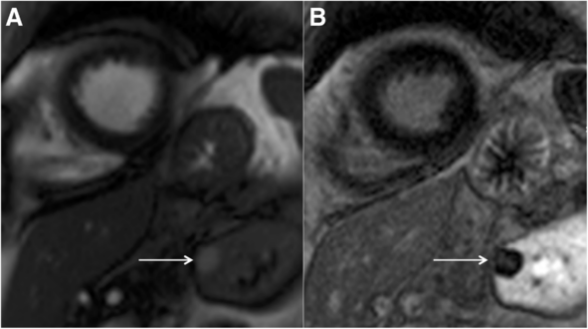
Short-axis steady state free precession cine image (**a**) revealing a high signal cyst in the visualized left kidney (solid white arrow). On the corresponding magnitude late gadolinium enhancement image (**b**) there is no enhancement of the cyst (solid white arrow) but diffuse enhancement of the surrounding normal renal parenchyma. This is a reassuring feature of renal cystic lesions

The challenge when faced with an incidental renal cystic lesion is to determine the likely malignant potential. The size and number of simple cysts are not usually clinical important in this regard. The latter is only really relevant in cases of excessive renal cysts, which may raise the possibility of polycystic kidney disease. The malignant potential of renal cysts can be predicted using the Bosniak classification [59] (Table 3). This classification is based on imaging findings on post-contrast CT but findings are similar at MRI [60] and therefore the classification can be loosely extrapolated to incidental cysts found at the time of CMR. The important message is that completely simple cysts have virtually no malignant potential but complex cysts with multiple septations and nodules as well as part-solid, part-cystic components are highly concerning for malignancy. Occasionally, renal cysts in the left kidney will be demonstrated in the field of view of LGE images (Fig. 17). These images may provide information about lack of cyst enhancement, which is a reassuring feature.

**Table 3:** The Bosniak renal cyst classification system – adapted from reference [59]

Bosniak I • Simple cyst o Hairline-thin wall, no septations, no calcifications, no solid components o Management: Nil o Malignant potential: Approximately 0 %	
Bosniak II • Minimally complex o May contain a few hairline-thin septa with no perceivable enhancement, fine calcification or short segment of thicker calcification in wall or septa, or uniformly high-attenuation lesions on CT <3 cm that are sharply marginated and do not enhance o Management: Nil o Malignant potential: Approximately 0 %	
Bosniak IIF • Minimally complex o Multiple hairline-thin septa, perceived septal enhancement but no measureable enhancement, minimal wall thickening, calcification may be thicker and nodular, no enhancing soft tissue components, or totally intra-renal non-enhancing high-attenuating renal lesions >3 cm o Management: Ultrasound or CT follow-up o Malignant potential: Approximately 5 %	
Bosniak III • Indeterminate o Thickened irregular or smooth walls or septa with measureable enhancement o Management: Partial nephrectomy or radiofrequency ablation o Malignant potential: Approximately 55 %	
Bosniak IV • Clearly malignant o All criteria of Bosniak III but also contain discrete enhancing soft tissue components independent of the wall or septa o Management: Partial or complete nephrectomy o Malignant potential: Approximately 100 %

Differentiating para-pelvic cysts from hydronephrosis can be challenging. The important ancillary findings that favour the latter include blunting of the renal calyces (Fig. 16b) and associated cortical atrophy (but this will only be present in longstanding obstruction). The presence of a concomitant dilated ureter, if demonstrated on the CMR images, also favours obstruction over para-pelvic cyst (Fig. 16b). It should be noted that para-pelvic cysts are more likely to be incidental and urinary obstruction is more likely to present with corresponding clinical and biochemical abnormalities.

### Further investigations

Before considering additional imaging, it is important to review any previous renal imaging. Stability in appearances over a long period of time is clearly a reassuring feature. Ultrasound is a readily available modality that can help further characterize incidental renal lesions in the first instance. If there is any concern of urinary obstruction, an urgent Urological opinion should be sought. It there are any concerns regarding possible malignant potential of renal cystic lesions or other renal masses, seek expert uro-radiological input and have a low threshold for urgent review at the Urology cancer multidisciplinary team meeting. A contrast enhanced CT study will be needed to definitively characterize the lesion and stage any potential disseminated metastatic disease.

## Spleen

The spleen is often best visualized in coronal anatomical imaging sequences, if performed. It may be incompletely imaged in axial dataset.

### Normal variant anatomy

**Fig. 18:**
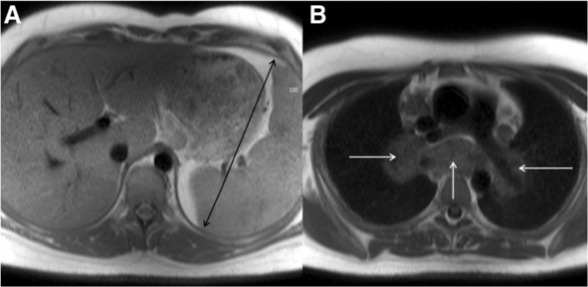
Axial black blood imaging of the upper abdomen (**a**) revealing splenomegaly, with maximal bipolar diameter of the spleen of 17.1 cm (solid black arrows). This was detected incidentally at the time of CMR. Scrutinizing the other available extra-cardiac images (**b**) revealed extensive mediastinal lymphadenopathy (solid white arrows), suggesting the splenomegaly was due to lymphoma in this patient

In adults, the normal size of the spleen is between 12 – 15 cm. Care should be taken to ensure measurements are performed in maximal bipolar dimension (Fig. 18a). Elevated height and increased body mass index are associated with larger splenic dimensions in otherwise normal spleens.

It is fairly common to encounter rounded, well-circumscribed soft tissue masses in the left upper quadrant in the vicinity of the spleen. These are invariably a focus / foci of accessory splenic tissue termed a splenunculus / splenuculi.

**Fig. 19:**
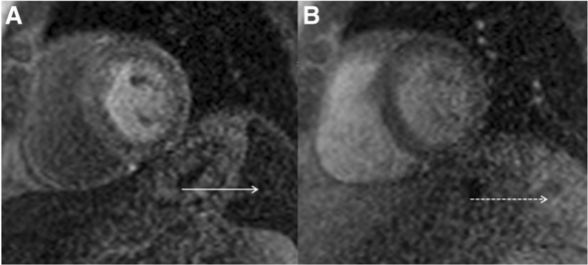
Real-time first pass myocardial perfusion at peak stress following administration of intravenous adenosine (**a**) revealing no discernible enhancement of the visualized spleen (*solid white arrow*). Real-time first pass myocardial perfusion at rest in the same patient (**b**) revealing normal diffuse enhancement of the splenic tissue in the field of view (*dashed white arrow*)

The normal physiology of the spleen should be understood by individuals who perform adenosine stress perfusion CMR. The spleen can down-regulate its function during ‘stress’ with intravenous adenosine which manifests as lack of enhancement, either assessed visually or quantitatively, on the stress images relative to the rest images (Fig. 19) [61]. In addition to heart and blood pressure changes and development of symptoms, the so-called ‘splenic switch-off’ sign may be another useful marker to ensure the patient has been adequately stressed during an adenosine perfusion CMR [61].

### Common pathology

Splenomegaly should be assessed as described. There are numerous causes of splenic enlargement. However, certain causes of splenomegaly are more likely to be present depending on the indication of the CMR. For example, in patients being assessed for iron loading, haematological disorders are the most likely cause of splenomegaly. Congestive splenomegaly may occur in right heart or biventricular heart failure. Viral pathogens may cause both splenomegaly and viral myocarditis. Amyloidosis and sarcoidosis are other disorders that may cause concomitant cardiac and splenic pathology.

Focal splenic lesions are uncommon. In the case of incidentally detected lesions, they most likely represent haemangiomata or the sequelae of previous trauma. An expert radiological opinion should be sought in any uncertain cases.

### Important imaging findings

When splenomegaly is discovered, the available images should be scrutinized for potential clues to the aetiology if not already clinical apparent (Fig. 18b).

As with the liver, it is important to assess the signal characteristics of the spleen. This can help distinguish between primary and secondary iron overloading conditions as explained in the *Liver, Biliary tree and Pancreas* section of this review.

### Further investigations

In the context of splenomegaly, further imaging is rarely additive and the underlying aetiology needs to be determined clinically. In the case of incidental focal splenic lesions, reviewing any previous imaging should be the first step. Ultrasound is often performed as a readily available and non-ionising radiation modality to further characterize splenic abnormalities. Discussion with a Radiologist about the necessity for further investigation is then advised.

## Adrenal glands

The adrenal glands are often visualized on the axial and coronal anatomical imaging performed at the beginning of a CMR study.

### Normal variant anatomy

On axial and coronal MR images, the right adrenal gland is located immediately posterior to the inferior vena cava and superior to the upper pole of the right kidney. It has a linear, inverted V or Y configuration [62]. The left adrenal gland is anteromedial to the upper pole of the kidney and posterior to the pancreas and has a triangular inverted Y or V configuration [62]. Normal adrenal glands range from 2 to 6 mm in thickness and from 2 to 4 cm in length [62].

### Common pathology

**Fig. 20:**
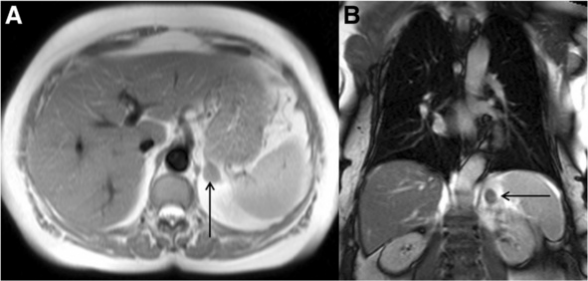
Axial black blood imaging (**a**) and coronal bright blood imaging (**b**) revealing an incidental left adrenal nodule (solid black arrow) detected at the time of CMR

**Fig. 21:**
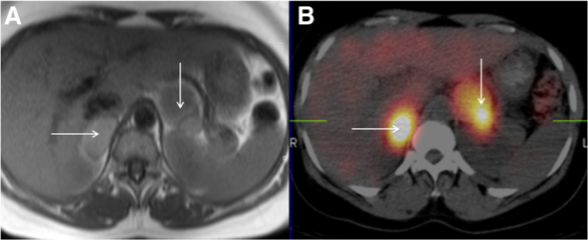
Axial black blood imaging (**a**) demonstrating abnormal soft tissue signal masses in the anticipated anatomical location of the adrenal glands (*solid white arrows*). These areas were confirmed to be highly metabolically active on metaiodobenzylguanidine (MIBG) study (**b**) confirming the diagnosis of bilateral phaeochromocytomas, which were the cause of this patient’s hypertension

Adrenal masses are occasionally encountered incidentally at the time of CMR (Fig. 20). The differential diagnosis includes primary and metastatic malignancy as well as benign entities. It should be appreciated that abnormal adrenal function can occur in anatomically normal appearing adrenal glands and biochemical correlation should be performed if there is any clinical suspicion. Biochemically active lesions are particularly important to consider in subjects being investigated with CMR for hypertensive heart disease as this may represent a treatable secondary cause of their hypertension (Fig. 21).

### Important imaging findings

When an incidental adrenal lesion is identified, the important clinical question is whether the abnormality represents a benign or malignant lesion. The biggest risk factor for malignancy in an incidental adrenal lesion is a history of known primary malignancy elsewhere; adrenal metastases are found in as many as 25 % of patients with known primary lesions [62]. Common primary sites of malignancy that frequently metastasise to the adrenals include lung, colon, breast and pancreatic tumours.

**Fig. 22:**
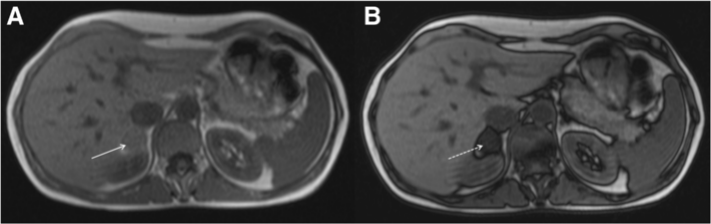
In-phase MRI image (**a**) showing a right adrenal lesion (solid white arrow). There is signal drop out (dashed white arrow) on the out-of-phase MRI sequence (**b**), confirming the presence of intracellular lipid and making the diagnosis of a benign lipid-rich adenoma

The documentation of macroscopic fat or MRI imaging features of intracellular lipid are reassuring signs that signify the lesion represents a lipid-rich benign adenoma. The lack of demonstrable fat means the lesion may still have malignant potential. If the adrenal entity is appreciated whilst the patient is still undergoing CMR, a simple additional in/out phase MRI sequence can be performed to try to distinguish benign from malignant disease (Fig. 22). This sequence relies on chemical shift artifact; signal loss on out-of-phase imaging relative to in-phase imaging confirms intracellular lipid and benignity (Fig. 22).

### Further investigations

Adrenal lesions can be characterized by adrenal protocol CT and the decision to use MRI or CT depends on local availability and the individual patient (ionizing radiation should be minimized wherever possible, particularly in the young).

## Bones

**Fig. 23:**
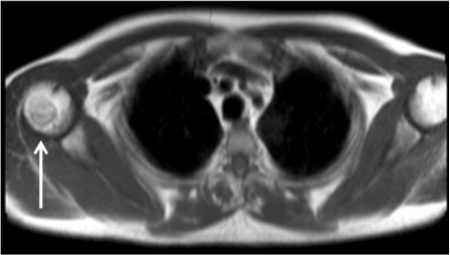
Axial black blood lesion demonstrating a bony lesion in the right humeral head (*solid white arrow*)

The thoracic rib cage, thoracic axial skeleton and proximal humeri (Fig. 23) are routinely imaged at the time of CMR. Familiarity with common and important osseous pathology is important for individuals reporting CMR images.

### Normal variant anatomy

**Fig. 24:**
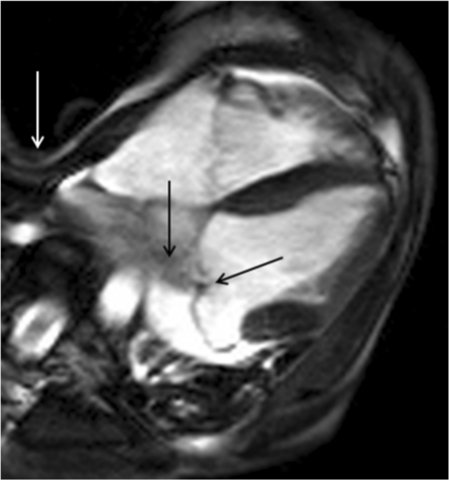
4-Chamber steady state free precession cine showing mitral valve prolapse with central coaptation defect and a jet of mitral regurgitation (*solid black arrows*). There is a marked pectus exacavatum chest wall deformity (*solid white arrow*). The unifying diagnosis in this case was Marfan’s syndrome

Pectus carinatum and pectus excavatum are chest wall deformities that are occasionally encountered. The latter is important to recognize as it may account for cardiac dextroposition and extreme dextroversion. It can also impinge the right ventricular free wall resulting in apparent regional wall motion abnormalities which are important to recognize, particularly in CMR studies being performed for suspected arrhythmogenic right ventricular cardiomyopathy / dysplasia. The presence of pectus excavatum (Fig. 24) and other spinal abnormalities, such as dural ectasia, may help support a unifying underling diagnosis of Marfan’s syndrome.

It should also be recognized that congenital heart disease is associated with congenital spinal abnormalities, such as kypho-scoliosis and hemi-vertebrae, and particular care should be taken when reporting congenital heart disease CMR for concomitant bony anatomical variations.

### Common pathology

**Fig. 25:**
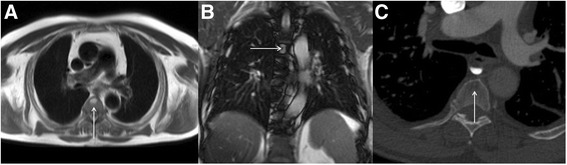
Axial black blood imaging (**a**) and coronal bright blood imaging (**b**) showing a high signal entity within a thoracic vertebral body, with no involvement of the posterior elements (*solid white arrows*). The patient had previously undergone a thoracic CT (**c**) and this confirmed the classic appearance of a benign thoracic haemangioma with central ‘polka dot’ sign (*white arrow*)

Lesions in the thoracic vertebral bodies are occasionally encountered. Incidental, benign vertebral body haemangiomas are a common cause of such lesions (Fig. 25). However, the bone is a common location for metastatic disease and bony metastases are an important differential to consider (Fig. 13c and e).

Other bony abnormalities may be demonstrated depending on the clinical indication of the CMR. For example, broken ribs are frequently encountered in subjects undergoing CMR following successful cardiopulmonary resuscitation.

Depending on the age of the subject undergoing CMR, degenerative spinal changes may be very frequently encountered. Prominent osteophytes should not be confused with sinister pathology. Degenerative vertebral body disc protrusions, also known as Schmorl’s nodes, are also common. They frequently have a calcific low T1 and T2 signal rim but are contiguous with vertebral body endplates.

### Important imaging findings

The key question when faced with a vertebral body lesion is: can benign disease be differentiated from malignant disease on the images available? Haemangiomas are vascular, fatty benign lesions. They appear bright on both T1 and T2 weighted images (Fig. 25a and b). Metastastic disease can be broadly divided into osteoblastic and lytic disease. The former usually return low T2 weighted signal but lytic lesions may return high T2 weighted signal. Importantly, both osteoblastic and lytic metastases usually return low T1 weighted signal, which helps distinguish from haemangiomas. Multiple lesions, abnormal surrounding soft tissue, prior malignancy, increasing age and/or smoking history are factors that would increase the index of suspicion for metastatic disease. If a potential metastatic lesion is identified, it is important to assess for associated vertebral body destruction or collapse and retropulsion of bony fragments towards the spinal canal. Benign lesions rarely extend into the posterior elements (vertebral pedicles, lamina or spinous processes). Posterior element involvement suggests the lesion may be unstable. Whenever a potential complication of metastatic spinal disease is noted, urgent oncological opinion is advised as urgent radiotherapy may be required.

Degenerative intervertebral disc disease often manifests as loss of intervertebral disc height. A degenerative disc should be low signal on T1 and T2 weight imaging. If there is high intra-disc signal on T2 weighted imaging, discitis needs to be considered. However, discitis usually does not present incidentally and constitutional symptoms are often present and there may be other imaging findings such as a local inflammatory soft tissue reaction.

**Fig. 26:**
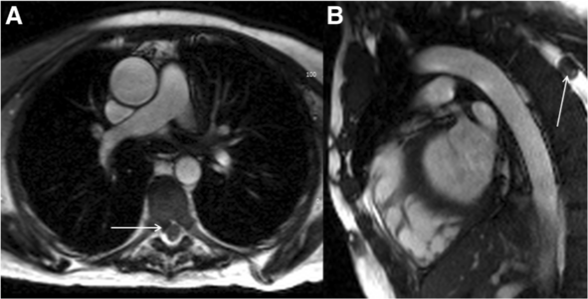
Axial steady state free precession cine image (**a**) and main pulmonary artery long-axis stack steady state free precession cine image (**b**) revealing an abnormal mass within the spinal canal (*solid white arrows*) detected incidentally at the time of CMR

Finally, it is good practice to review the spinal canal and cord as occasionally incidental central neuroaxis lesions will be identified (Fig. 26).

### Further investigations

Previous comparative CT imaging can be very useful to reassure the interpreter that the suspected lesion is in fact a benign, incidental haemangioma. These lesions are characteristically well-defined, low-attenuation areas with central ‘polka dot’ appearance due to prominent bony trabeculations that can only be visualized with the higher spatial resolution that CT affords (Fig. 25c). Previous CT scans may also identify a putative primary lesion in the case of suspected metastatic disease.

## Conclusion

**Fig. 27:**
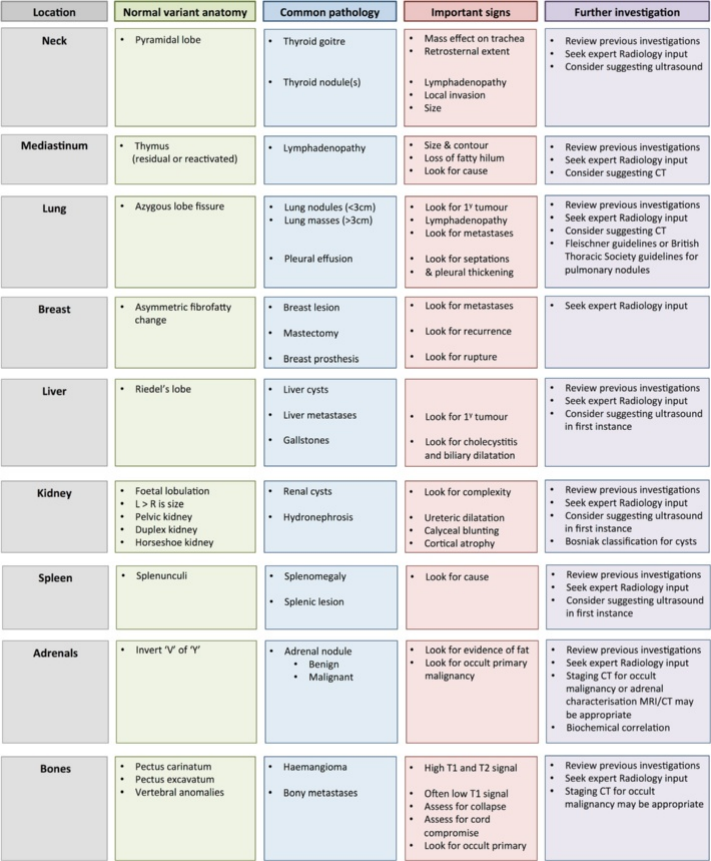
Normal variant anatomy, common pathology, important imaging signs and potential further investigations for extra-cardiac findings discovered at the time of CMR

A significant amount of the neck, thorax and upper abdomen is imaged at the time of routine clinical CMR. Extra-cardiac findings are common and an important proportion of these findings are clinically relevant. Individuals who report CMR have a professional and ethical duty to be adequately trained in the interpretation of extra-cardiac findings or seek the opinion of radiology colleagues who are so. We have reviewed normal anatomical variants that may mimic pathology, common extra-cardiac findings and important imaging signs not to miss when reporting extra-cardiac findings at the time of CMR (Fig. 27). Correctly interpreting extra-cardiac findings is beneficial to the patient and can prevent unnecessary over-investigation whilst ensuring indeterminate or potentially important lesions are investigated appropriately. The diseases affecting the heart also often have systemic effects and identification of certain extra-cardiac findings can help with the interpretation of the primary cardiac question. Our review only touches upon the imaging findings of a variety of important diseases in many different organ systems and should not be used as a replacement to seeking expert Radiological input where there is uncertainty. It is beyond the scope of this review to discuss and determine the clinical significance of extracardiac findings at CMR. Our parting piece of advice is that the next best investigation is usually reviewing the last imaging investigation the patient underwent.

## Abbreviations

CMR: cardiovascular magnetic resonance
MRI: magnetic resonance imaging
FNA: fine needle aspiration
CT: computed tomography
LGE: late gadolinium enhancement
